# Comparison of metabolites and variety authentication of *Amomum tsao-ko* and *Amomum paratsao-ko* using GC–MS and NIR spectroscopy

**DOI:** 10.1038/s41598-021-94741-0

**Published:** 2021-07-26

**Authors:** Huiwei Qin, Yuanzhong Wang, Weize Yang, Shaobing Yang, Jinyu Zhang

**Affiliations:** 1 Medicinal Plants Research Institute, Yunnan Academy of Agricultural Sciences, Yunnan, 650200 Kunming China; 2 College of Traditional Chinese Medicine, Yunnan University of Chinese Medicine, Yunnan, 650500 Kunming China

**Keywords:** Biochemistry, Plant sciences

## Abstract

*Amomum tsao-ko*, as an edible and medicinal variety, has been cultivated for more than 600 years in China. Recently, two cultivars, *A. tsao-ko* and *Amomum paratsao-ko*, were found in *A. tsao-ko* planting area. The two cultivars are often confused because of the similar phenotype and difficult to distinguish through sensory judgment. In this study, the non-targeted gas chromatography-mass spectrometry (GC–MS) metabolomics combined with near-infrared spectroscopy (NIRS) were used for dissecting the two cultivars with phenotypic differences. According to principal component analysis (PCA) loading diagram and orthogonal partial least squares discriminant analysis (OPLS-DA) S-plot of the metabolites, the accumulation of major components including 1,8-cineole, α-phellandrene, (E)-2-decenal, (−)-β-pinene, (E)-2-octenal, 1-octanal, D-limonene, and decanal, were present differences between the two cultivars. Seven metabolites potential differentiated biomarkers as β-selinene, decamethylcyclopentasiloxane, (E,Z)-2,6-dodecadienal, (E)-2-hexenal, (E)-2-decenal, isogeranial, 1,8-cineole and β-cubebene were determined. Although *A. tsao-ko* and *A. paratsao-ko* belong to the same genera and are similar in plant and fruit morphology, the composition and content of the main components were exposed significant discrepancy, so it is necessary to distinguish them. In this study, the discriminant model established by GC–MS or NIRS combined with multivariate analysis has achieved a good classification effect. NIRS has the advantages of simple, fast and nondestructive and can be used for rapid identification of varieties and fruit tissues.

## Introduction

*Amomum tsao*-*ko* Crevost et Lemaire is a perennial evergreen plant of Zingiberaceae, mainly distributed in southeastern China as well as parts of Vietnam and northern Laos^1^. Its fruit named “Cao Guo” in Chinese, which is usually added to food as a flavoring agent to enhance the unique flavor of food and treats various infections and diseases of the digestive system as a traditional Chinese medicine, such as pneumonia, malaria, indigestion and diarrhea^2^. *A. tsao-ko* contains a large amount of volatile oil including monoterpenes, sesquiterpenes, fatty acids, which attracts wide attention due to various biological activities, such as antioxidant, anti-inflammatory, anti-tumor, antibacterial^3,4^.

*A. tsao-ko* has been cultivated in Yunnan more than 300 years and has become an economic crop in many areas of the province at present. Planting area and output of Yunnan account for more than 90% in China, of which Nujiang is the main producing area^5^. When the research group conducted a survey of *A. tsao-ko* in Yunnan, two cultivars, *A. tsao-ko* with yellow flower, *A. paratsao-ko* with white flower, were found in the planting area. Interestingly, the two cultivars were only five meters apart. Although flower colors were different, the fruit shapes were similar, leading to difficulty in distinguishing according to macroscopic characteristics. As a result, they were often harvested confusingly and sold in markets by farmers. Traditional methods of identifying the source and quality of *A. tsao-ko* include simply observing color, shape and size, and aroma, which lack accuracy and versatility because they require experienced professionals and largely considered subjective judgments. In recent years, scientists had conducted extensive studies on germplasm resources of *A. tsao-ko* using microsatellite marker^6^, rbcL gene sequence^7^, chemical fingerprint^8^ and so on, providing a lot of the basis for determining source and classification of *A. tsao-ko* to a certain extent. As far as we know, the above studies are mostly focused on a single variety, but there are few comparative studies on the *A. tsao-ko* and *A. paratsao-ko*.

Modern analytical techniques such as GC–MS and NIRS can be used as reliable tools to evaluate and differentiate plant materials of different varieties, origins and processing procedures. GC–MS has also been widely used to evaluate quality of raw materials and final products in *A. tsao-ko*. Sim et al. used GC–MS to analyze *A. tsao-ko* and *Amomum subulatum* and found that there were 28 main constituents with varying degrees of change^8^. NIRS is often applied in quality evaluation because of the simple operation and minimum sample preparation. López-Hidalgo et al. used NIRS and MS-based approaches to successfully distinguish different traits of acorns and found that there were no correlations between chemical composition and morphometry^9^.

Metabolomics simultaneously detects hundreds of metabolites, which is widely devoted to the study of plant metabolites, biomarker screening and identification of medicinal and edible materials and so on^10–12^. According to research reports, flavonoids and anthraquinones are related to the color change of *Fagopyrum tataricum L.* Gaertn. seeds, meanwhile, flavonoids also contribute to significant differences in the shape of the seeds^13^. Plant organogenesis and species communication are also regulated under metabolites signals^14^. Studies have shown that excessive accumulation of trehalose in *Arabidopsis thaliana* may lead to growth inhibition, early flowering and increase shoot formation^15^. Phenolic chlorogenic acid has significant antibacterial activity, which considered to be an important barrier for plants to resist biotic and abiotic damage. The phenotypic differences of *A. tsao-ko* and *A. paratsao-ko* provide clues to study their metabolites and environmental adaptability. Volatile oil is not only the main active component in the two cultivars, but also the secondary metabolites of plants, which is a great significance of plants to defend themselves against dangerous environments, disease and insects, and competition between plants^16^. For example, plants release volatile terpenoids to attract natural enemies after being gnawed by herbivorous insects to form indirect defenses, and release fragrance to induce insect pollination, spawning, host orientation to form mutually beneficial relationships with other biological populations^17^. To the human body, most of them, such as terpenoids, alkaloids and aromatic compounds, have strong biological activity and special medical value.

In this study, we attempted for the first time to describe a comparable second metabolic profile in *A. tsao-ko* and *A. paratsao-ko* through a non-targeted GC–MS metabolomics strategy, and quickly identified the two varieties using NIRS. The subjects included basic morphological parameters, differential metabolites, and characteristic spectral regions.

## Materials and methods

### Fruit samples

Both of *A. tsao-ko* and *A. paratsao-ko* belong to the genus *Amomum* Roxb in the Zingiberaceae. They are difficult to distinguish due to the similar shape and color of the dried fruits and the similar spicy aroma. For a long time, the two varieties have been used confusedly in the medicinal and spice markets. The differences in morphological characteristics and chemical composition of plants are influenced significantly by multiplex growth environment, such as origin, altitude, rainfall, etc. Fortunately, *A. tsao-ko* (HH) and *A. paratsao-ko* (BH) were collected in the same growth status and adjacent position (about 5 m apart) from Nujiang of Yunnan, which reduced the impact of some ecological factors. The samples were identified as the fruits of *A. tsao-ko* Crevost et Lemarie and *A. paratsao-ko* S. Q. Tong et Y. M. Xia of Zingiberaceae by Dr. Jinyu Zhang (Medicinal Plants Research Institute, Yunnan Academy of Agricultural Sciences, Kunming, China). The samples dried were stored in a cool and ventilated place until metabolite analysis was carried out. Two fruit tissues, the pericarp and the seed, were tested separately, and divided into the pericarp of *A. paratsao-ko* (BH-P), the seed of *A. paratsao-ko* (BH-S), the pericarp of *A. tsao-ko* (HH-P), the seed of *A. tsao-ko* (HH-S) (each tissue site in three biological replicates). The study's authors promisethe use of plants in the present study complies with international, national and/or institutional guidelines.

### Gas chromatography-mass spectrometry (GC–MS) analysis

Solid phase micro-extraction (SPME) separation technology was used as the pretreatment method of the sample. The samples were added to a 20 mL headspace bottle, sealed with a cap, and then extracted were analyzed using a CTC trinity automatic sampler with extraction head (50/30 µm DVB/CAR on PDMS). The sample was shaken for 15 min at 250 rpm and then extracted for 30 min at 50 °C. Resolution time was 5 min, and cycle time was 50 min. GC–MS was carried out on an Agilent Technologies 7890B-5975B GC–MS unit with DB-wax Chromatographic column (30 m × 0.25 mm, 0.25 µm thickness). Chromatographic was set as follows: inlet temperature was 260 °C, helium gas was used as carrier gas, and constant flow rate was 1 mL/min. Temperature program was as follows: gradually increased to 40 °C and held for 5 min, and then increased to 220 °C at a rate of 5 °C/min, and reached a final temperature of 250 °C at a rate of 20 °C/min and held for 2.5 min. Mass spectrometry condition was as follows: the interface temperature was 260 °C, the ion source temperature was 230 °C, the quadrupole temperature was 150 °C, and the electron ionization (EI) mode was 70 eV. The scan range was 40–800 m/z in full scan mode.

The mass spectrometry data obtained from GC–MS was compared with the standard mass spectrometry database (National Institute of Standards and Technology Library 14.0 and the NEW Wiley 9 mass spectra library database) and the published literature to obtain a dataset containing peak intensity, retention time, and sample information. The original data were transformed into CDF format (NetCDF) using the Agilent GC/MS 5975 Data Analysis software and processed using the XCMS software (v.1.36.0; www.bioconductor.org). The processes of nonlinear retention time alignment, baseline filtration, peak identification, matching, and integration were included. The dataset was normalized using the total area of the peaks in each sample.

### Near-infrared spectroscopy (NIRS) analysis

NIRS analysis of samples were measured on a Antaris II Fourier transform near infrared (FT-NIR) spectrometer (Thermo Fisher scientific, USA). HH and BH were ground to fine powder and pass a 100 mesh sieve. The samples were loaded into a NIRS sample cup for measurement. Each sample was repeated 3 times, and the average spectrum was taken. The sample was scanned in 64 with a spectral resolution of 8 cm^−1^ and a scanning range of 12,000–4000 cm^−1^.

### Statistical analysis

The metabolites data underwent logarithmic transformation and normalization of auto‐scaling to enhance Min–Max Normalization and normality. Hierarchical cluster analysis (HCA) of the metabolites was carried out on R software to study the variations of HH and BH. The heatmap and histogram to display the experimental data structure were drawn by R-3.2 software (www.r-project.org) and Sigmaplot-10.0 (sigmaplot.en.softonic.com), respectively. Principal component analysis (PCA) loading diagram and orthogonal partial least squares discriminant analysis (OPLS-DA) were used to visualize the metabolic differences between cultivars after unit variance scaling and mean centering. The differential metabolites with univariate statistical significance were extracted (P < 0.05 and VIP > 1.0). Variable influence on projection (VIP) is a widely used method for screening important variables based on model, and values greater than 1 are generally considered to be important variables for classification^18^. Here, we screen for characteristic chemical composition and spectrum based on this value. Metabolites were mapped onto biochemical pathways according to annotation in Kyoto Encyclopedia of Genes and Genomes (KEGG) (www.kegg.jp).

Raw NIRS data contained vast noise and interference information. Common preprocessing methods as first derivatives (FD), multiplicative scatter correction (MSC) and Savitzky-Golay (SG) were applied to preprocess the original spectral data. PCA and partial least squares discriminant analysis (PLS-DA) were used to classify and screen the characteristic spectra using the dataset comprised of pretreated spectra to achieve better calibrations. The veracity of a model was evaluated in terms of some statistical parameters including the goodness of fit (R^2^) and the goodness of prediction (Q^2^). Briefly, R^2^ indicates the cumulative interpretation ability and Q^2^ demonstrates the cumulative prediction ability of the present model according to cross-validation, a good model should have a higher R^2^ and Q^2^ (close to 1). Origin 8.0 software (origin.en.softonic.com) was used to draw average NIRS and feature variable spectra. Multivariate statistical model was worked using SIMCA-P^+^ 14.1 (Umetrics, Sweden) (www.sartorius.com).

## Results and discussion

### Analysis of metabolites

As shown in Fig. S1, the fruits were present differences in color and size. The fruit of *A. tsao-ko* (HH) was large and brown, while the fruit of *A. paratsao-ko* (BH) was small and reddish-brown. But the dried fruits are the same color, all brown, and difficult to identify by the senses. The abundant phenotypic differences among cultivars may be reflected in the changes of metabolites, which is also an important reason for the analysis of metabolites. In order to compare the metabolite data of different orders of magnitude, 82 metabolites (Table S1) were selected from 222 metabolites detected in the GC–MS for this combinatorial analysis after data preprocessing such as exclusion of systematic errors. These metabolites were mainly volatile organic compounds of isomers, with the largest group contained 40 terpenoids accounting for 48.8% of the total metabolites, and the second largest group had 31.7% of the total identified metabolites: 26 aliphatic metabolites, followed by 5 aromatic groups (6.10%) and some other compounds (13.4%).

**Figure 1:**
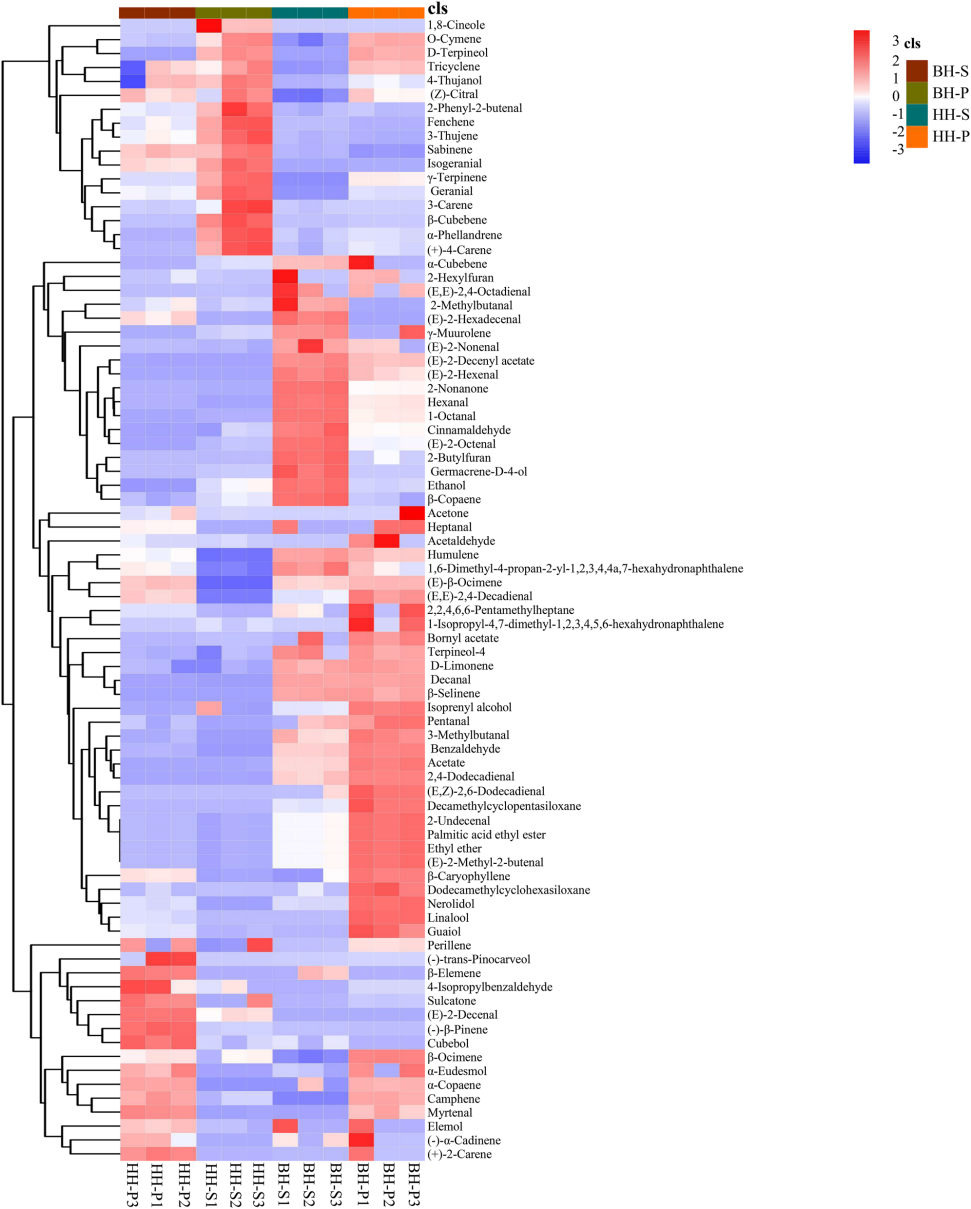
Heatmap clustering of the metabolites among HH and BH. BH-P represents the pericarp of *A. paratsao-ko*, BH-S represents the seed of *A. paratsao-ko*, HH-P represents the pericarp of *A. tsao-ko*, HH-S represents the seed of *A. tsao-ko*, the same as below.

Subsequently, the metabolites were visualized on a heatmap (Fig. 1) using HCA to capture the maximum number of metabolites that could change between each group. Hierarchical cluster analysis based on relative levels across samples made metabolites clusters among cultivars, and the differences in the relative content of metabolites were also clearly shown. Among them, 25 metabolites (30.5%) were upregulated in the pericarp of *A. tsao-ko* (HH-P) and 18 metabolites (22.0%) were upregulated in the seed of *A. tsao-ko* (HH-S). However, there were 46 upregulated (56.1%) metabolites in the pericarp of *A. paratsao-ko* (BH-P) and 30 upregulated metabolites (36.6%) in the seed of *A. paratsao-ko* (BH-S). The research reported that 75.0% differences in plant metabolites are caused by genetic factors, and plant with same biological origin have stable morphological characteristics and clinical efficacy^19^. Biological populations with the same genetic background will undergo changes in metabolism and resistance due to changes in environmental factors, resulting in phenotypic changes. This indicated that the production and distribution of plant metabolites are usually specific to species, organs, tissues, growth stages and environments. In this study, the relative contents of terpenoids in the tissues were significantly changed, followed by aliphatic metabolites and other metabolites were relatively stable. The volatile chemicals produced by plants including a wide variety of short-chain alcohols, aldehydes, ketones, esters, aromatic phenols, and lactones, as well as mono- and sesquiterpenes, which are major constituents of volatile organic chemicals produced in response to wounding and insect attack^20^. There was a research report that aliphatic and alkaloid products will highly enrich during traumatic stress^21^. The monoterpenes such as ( +)-sabinene and α- and β-thujone have been shown to resistance pathogens and herbivores in the western redcedar foliage^22^. BH is brightly colored and more attractive to insects. And the number of fruits on BH plant is less than HH plant, resulting in it may be more vulnerable when faced with the same external threat. Plants protect themselves through changing their physiology, investing more metabolic flux into defensive metabolism when under stress, such as producing secondary metabolites to help maintain dynamic balance and health. It has been reported that essential oils containing (E)-β-ocimene may have a positive effect on the defense of *Schinus terebinthifolius*^23^. β-Caryophyllene promotes cell proliferation and migration and wound healing through a variety of ways and mediates analgesic effects to relieve pain^24^. β-Ocimene is usually induced from vegetative plant tissues after the plant invaded and regulates plant interactions with parasites and herbivorous predators, which is often used as chemical indicators for attracting natural enemies of herbivorous insects in a variety of plants^25^. Studies have found that volatile signals, such as cis-jasmone, generated in plants attacked by herbivores, also induced β-ocimene emissions in undamaged neighbors to participate in defense^26^. In addition to its impacts on insects, β-ocimene mediates the expression of several genes related to plant defense through activating the jasmonic acid signaling pathway, suggesting the compound plays an active role in indirect insect defense and plant-to-plant communication^27,28^. Unsaturated aldehydes such as (E,E)-2,4-decadienal produced by diatoms during cell damage have an invisible inhibitory effect on the reproduction of herbivorous crustaceans^29^. Interestingly, these metabolites are also highly expressed in BH, which may be partly related to the defense mechanism of fruits. In the field investigation, it was found that HH and BH were usually cultivated under alpine forests, and most farmers adopted the method of "heavy planting and light managing" to make them grow naturally. So those plants were always in a state of "imitating wild". The deep forest environment is complex and diverse, so we speculate the reason for this difference may be the joint action of genetic characteristics and growth environment. Studies have reported that there was a phenomenon of removing the pericarp and only selecting the seed as the use part of HH^2^. Our results showed that the pericarp also has a high chemical composition and can be comprehensively utilized to increase the added value rather than discarded in the actual production and processing.

**Figure 2:**
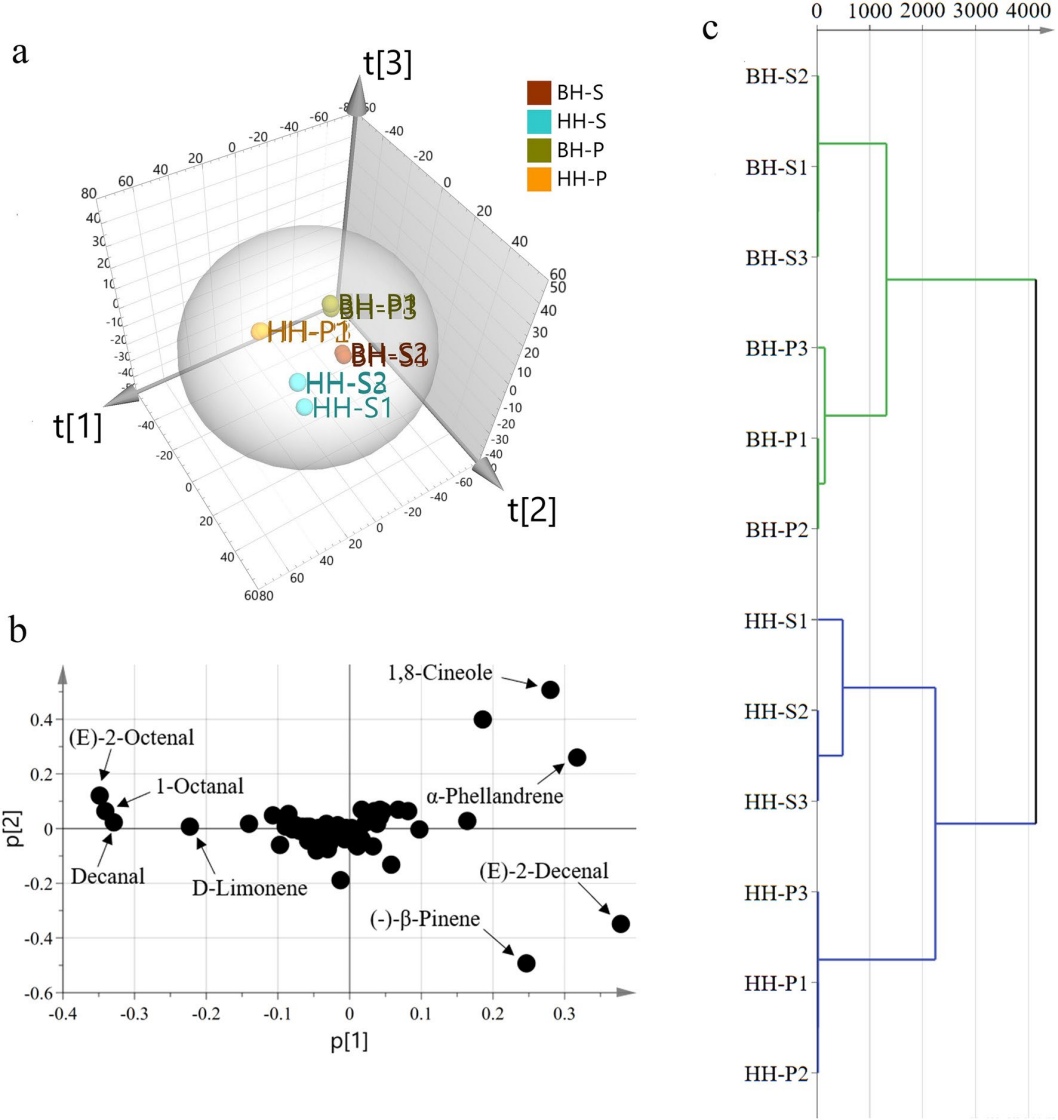
PCA 3D score plot (**a**) and loading plot (**b**) from the metabolites, showing metabolites and their assignments. Hierarchical cluster analysis among varieties and tissues (**c**).

For displaying data structure and species-specificity, the dimensionality of the data was reduced to mapping and visualization the groups of samples. Regarding the metabolites PCA 3D scores plot (Fig. 2a), the three principal components explained 91.5% of the overall variance of the metabolite profiles, 50.5%, 26.7% and 14.3% for PC1, PC2 and PC3. The R^2^ value was 0.982 and the Q^2^ value was 0.916. HH and BH showed clear separation, and the pericarps and seeds were also independent groups. The tight grouping of each biological replicate indicated the model validity, as well as technical variability and low biological. The loading plot (Fig. 2b) exposed metabolites that contributed to tissues and fruits segregation pattern^30^. 1,8-Cineole and α-phellandrene were abundant in HH-S, however, (E)-2-decenal and (−)-β-pinene were more abundant in HH-P. Additionally, the PCA loading plot also revealed the metabolites, (E)-2-octenal, 1-octanal, D-limonene and decanal, were abundant in BH-P and BH-S. Studies have shown that 1,8-cineole, citral, α-phellandrene, α-terpineol, γ-terpinene, β- and α-pinene, and nerolidol were main components of HH^4,31^, indicating the major components play an important role in the identification model. As shown in the HCA plot (Fig. 2c), HH and BH were strictly divided into two broad categories. The clustering distance between cultivars was larger than individual tissues, revealing that the differences were mainly reflected in cultivars rather than individual tissues.

### Analysis of differential metabolites

**Figure 3:**
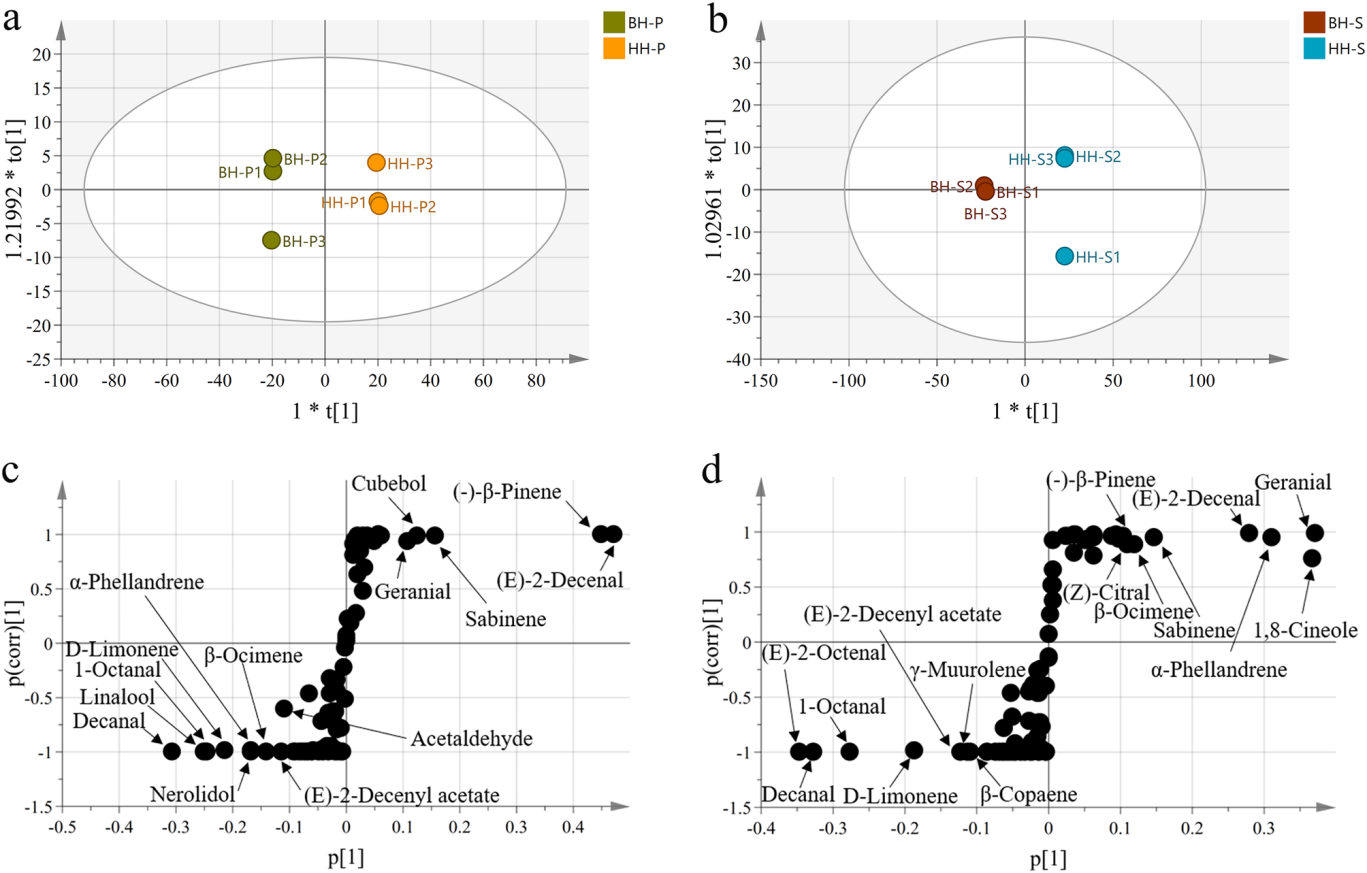
OPLS-DA score plot derived from modelling the metabolites in the pericarps and seeds of HH and BH (**a**,**b**). The S-plot showed the covariance t[1] and the correlation to[1] of the variables of the discriminating components in OPLS-DA model (**c** and **d**).

OPLS-DA maximize class separation through a supervised priori knowledge, rather than interpreting variations within a set of samples. The model was applied to assess the effects of metabolites in different varieties and highlight the abundance of metabolites in the pericarps and seeds^30^. The two models score diagram (Fig. 3a,b) showed quite effective separation among varieties and tissues with good predictive and excellent classification ability (The pericarps; R^2^ 100% and Q^2^ 99.9%, the seeds; R^2^ 99.9% and Q^2^ 99.8%). The two OPLS-DA models achieved 100% classification accuracy in the condition of without overfitting through 200 iteration tests (R^2^Y < 0.4), indicating that the model was applicative (Fig. S2). The inspection of S-plot, a tool generated through plotting the correlation against the covariance, revealed pivotal metabolites responsible for noteworthy distinct variation among the pericarps and seeds (Fig. 3c,d). As far as the pericarps is concerned, α-phellandrene (1.96-fold), D-limonene (5.64-fold), β-ocimene (1.38-fold), 1-octanal (23.6-fold), linalool (5.60-fold), decanal (24.9-fold), (E)-2-decenyl acetate (78.1-fold), acetaldehyde (6.53-fold) and nerolidol (3.79-fold) were differential markers enriched in BH-P. Nevertheless, sabinene (4.00-fold), geranial (1.21-fold) and cubebol (180-fold) were more accumulated in HH-P, (−)-β-pinene (17.6%) and (E)-2-decenal (19.5%) were only accumulated in HH-P. Likewise, the S-plot also disclosed for a richer (E)-2-octenal (4.93-fold), 1-octanal (19.7-fold), decanal (244-fold), D-limonene (7.07-fold), γ-muurolene (4.59-fold), and β-copaene (3.61-fold) in BH-S, (E)-2-decenyl acetate (1.69%) was only expressed in BH-S. On the contrary, α-phellandrene (3.86-fold), β-ocimene (1.75-fold), (Z)-citral (1.56-fold), (−)-β-pinene (3.55-fold) and sabinene (3.13-fold) were abundant in HH-S. 1,8-Cineole (19.3%), (E)-2-decenal (8.72%), and geranial (15.5%) were only accumulated in HH-S. Plant secondary metabolites are mostly a class of compounds with unique functions and biological activities^32^. Previous studies have reported that BH and HH have similar effects and are often used together as substitutes for each other^33^. In this study, it was found that the compositions and accumulative contents of the main components showed significant discrepancy between the two varieties, which might lead to different biological activities.

**Figure 4:**
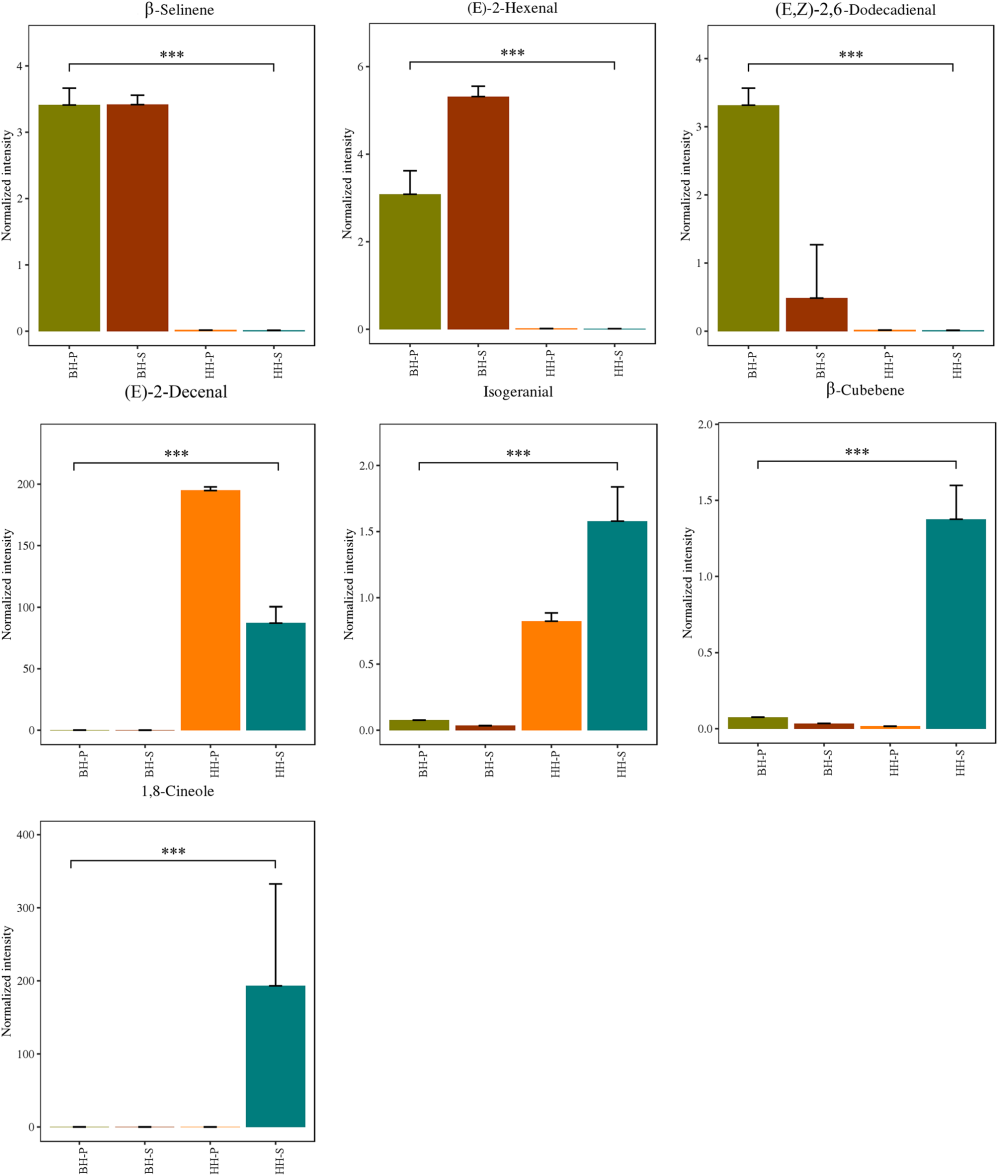
Specific expression of metabolites bar chart among HH and BH.

A total of 13 metabolites (Table S2) specifically expressed between varieties were eventually identified from the 82 metabolites, and 7 metabolites potential differentiated biomarkers were screened (Fig. 4). Among them, 3 metabolites, β-selinene, (E,Z)-2,6-dodecadienal and (E)-2-hexenal, were expressed only in BH. The other 4 metabolites, (E)-2-decenal, isogeranial, 1,8-cineole and β-cubebene, were expressed only in HH, whereas 1,8-cineole and β-cubebene were expressed only in HH-S. It has been reported that there are significant differences in the shape and size and a relationship between the content of volatile oil and fruit phenotype of HH among different populations^31^. Some scholars extracted the volatile oil from HH by modified-solvent free microwave extraction (M-SFME)^4^, ultrasonic extraction^34^ and hydrodistillation^35^. The results revealed that chemical composition of volatile oil was very complex. Chemical composition and relative content were different with different extraction methods and regions, but the main components of volatile oil were similar, which have also been confirmed in our results. Huang et al. identified 47 components from HH and 36 components from BH, and found a total of 20 chemical components in common between the two varieties, but the main chemical components were different, which was consistent with our research results^36^. In this study, it was found that HH and BH shared 69 metabolites, which may be related to analytical methods and samples.

### Analysis of metabolic pathway of the different metabolites and specific metabolites

Enrichment analysis showed that the different metabolites and specific metabolites were mainly concentrated in C10 isoprenoids, fatty aldehydes, ketones, C15 isoprenoids and sesquiterpenoids (Fig. S3). Isoprenoid compounds might be particularly relevant in adapting plants to adverse external conditions by serving as additional and/or alternative protection mechanisms and play vital roles in all living systems. Fatty aldehydes are important components of the cellular lipidome and serve intermediates in the interconversion between fatty acids and fatty alcohols. Fatty aldehydes are crucial substances that mediate a wide range of vital physiological functions, particularly lipid peroxidation. Ketones have long been considered to be abnormal and unpopular by-products of incomplete oxidation of fat. They only recognized as normal circulating metabolites in the early twentieth century, and intense ketogenic diets were found to be highly effective in treating drug-resistant epilepsy in children. Circulating ketones can replace glucose as the brain's primary fuel during hyperketonemia. There is growing evidence of mitochondrial dysfunction and reduced bioenergy efficiency in the brains of sufferer with Parkinson's disease and Alzheimer's disease, since mitochondria effectively use ketones to generate ATP and may also help protect vulnerable neurons from free radical damage^37^.

In this study, a total of 9 metabolites were found to have corresponding KEGG annotations (Table S1). Among these metabolites, D-limonene is involved in the monoterpenoid biosynthesis, biosynthesis of terpenoids and steroids and limonene and pinene degradation. D-Limonene can be synthesized from (+)-α-pinene, β-pinene, nerolidol, and (R)-linalool. D-Limonene derivatives such as α-pinene, perillyl alcohol, terpineol, carvone and carveol also have important application value. Acetaldehyde is a natural substance that exists widely in plant cells and tissues and is involved in multiple metabolic pathways such as glycolysis/gluconeogenesis, phenylalanine metabolism, phosphonate and phosphinate metabolism, pyruvate metabolism, and degradation of aromatic compounds. 1,8-Cineole is formed by (−)-α-terpineol and geranyl diphosphate and is a precursor of 6-endo-hydroxycineole and oxidized flavodoxin. 1,8-Cineole is involved in the monoterpenoid biosynthesis and inflammatory mediator regulation of TRP channels. Menthol, 1,8-cineole and icilin interact with TROM8 channel to affect the tryptophan metabolism. (−)-β-Pinene is produced from by geranyl diphosphate by cyclization of geranyl-diphosphate diphosphate lyase, which is involved in the monoterpenoid biosynthesis. Nerolidol is a naturally occurring sesquiterpene alcohol and is synthesized as an intermediate in the production of (3E)-4,8-dimethy-1,3,7-nonatriene (DMNT), which is an herbivore-induced volatile that protects plants from herbivore damage. Nerolidol is involved in the biosynthesis of terpenoids and steroids and sesquiterpenoid and triterpenoid biosynthesis. Geranial and (Z)-citral are downstream products of nerolidol, and geranial can be synthesized by (Z)-citral. Geranial and (Z)-citral are both involved in the biosynthesis of terpenoids and steroids and geraniol degradation. β-Selinene is formed by trans, trans-farnesyl diphosphate and is involved in the sesquiterpenoid and triterpenoid biosynthesis. (E)-2-Octenal is generally considered as a plant secretion with defensive properties and is involved in the prodigiosin biosynthesis. The reaction of (E)-2-octanal and pyruvate can produce (S)-3-acetyloctanal, octanal, 1-octanol, trans-2,4-decadienal, 2-octenoic acid, etc., these compounds are all downstream products of (E)-2-octenal. In general, the different metabolites and specific metabolites were primarily involved in the monoterpenoid biosynthesis, biosynthesis of terpenoids and steroids, and sesquiterpenoid and triterpenoid biosynthesis. Terpenoids are natural products with various structural characteristics produced by isoprene biosynthesis pathway. Isoprenoid cyclase is the key enzyme leading to the diversity of isoprenoid. On various occasions, these polypentenyl bisphosphates undergo a series of cyclists to generate the parent skeletons of monoterpenes (C10), sesquiterpenes (C15) and diterpenes (C20), triterpenoid (C25), followed by various modifications such as hydroxylation, methylation, and glycosylation to produce thousands of different isoprene metabolites. Recent studies have shown that almost all isoprene compounds such as monoterpenes, diterpenes and carotenoids are synthesized by the methylerythritol phosphate (MEP) pathway in plastids, while the mevalonate (MVA) pathway is responsible for the biosynthesis of sesquiterpenes and triterpenes^38^. The two pathways of terpenoid biosynthesis also communicate with each other. Studies found that intermediates generated from the MVA and MEP can transport between cytoplasm and plastids, which has been found in *Arabidopsis thaliana* and tobacco and other plants^38–40^. Meanwhile, these terpenoids can be transformed into each other under certain conditions, such as limonene and linalool can be converted into α-terpineol under the action of enzymes^41^.

When metabolites are involved in metabolic pathways, the final substances expressed lead to differences in plant species. For example, 1,8-cineole is rich in specific expression of HH, which can synthesize 2-exo-hydroxy-1,8-cineole and oxidized flavodoxin, etc. Studies have reported that 1,8-cineole induces arrest and senescence in HepG2 cells by oxidative stress and multiple signaling pathways, thereby inhibiting the growth of cancer cells^42^. A recent study found that 1,8-cineole can effectively bind the main viral proteinase (Mpro/3CLpro) to inhibit the coronavirus reproduction process, and it may have potential therapeutic potential as an inhibitor of COVID-19 Mpro^43^. In addition, 1,8-cineole has been widely reported for the anti-inflammatory, antioxidant, antibacterial, insecticidal and other activities^44^. Although the phenotypes of these two varieties were similar, they had their own characteristics, and their metabolic pathways and metabolic energy were still different. HH had larger fruit, higher yield and large seed capacity, but the accumulation of metabolites was less in its pericarp. It is speculated that HH carried more seeds and needed more nutrients to meet the requirements of metabolism, leading to the transfer of metabolic energy to seeds. Plant resources available for reproduction are limited and there may be trade-offs between two or more organs competing for the same resource library. The role of pericarps is usually to protect seeds and help them spread. More highly expressed metabolites in the pericarp of BH might have better resistance to the complex environment or be more vulnerable to threats in the same environment so that the metabolic energy transferred to the pericarp. In the face of the same environmental changes, HH and BH showed different metabolic mechanisms and reactions with different metabolites, and the biological activity and value of the two varieties also changed, which may also be due to the different gene expression profiles.

### Interpretation of NIRS feature

**Figure 5:**
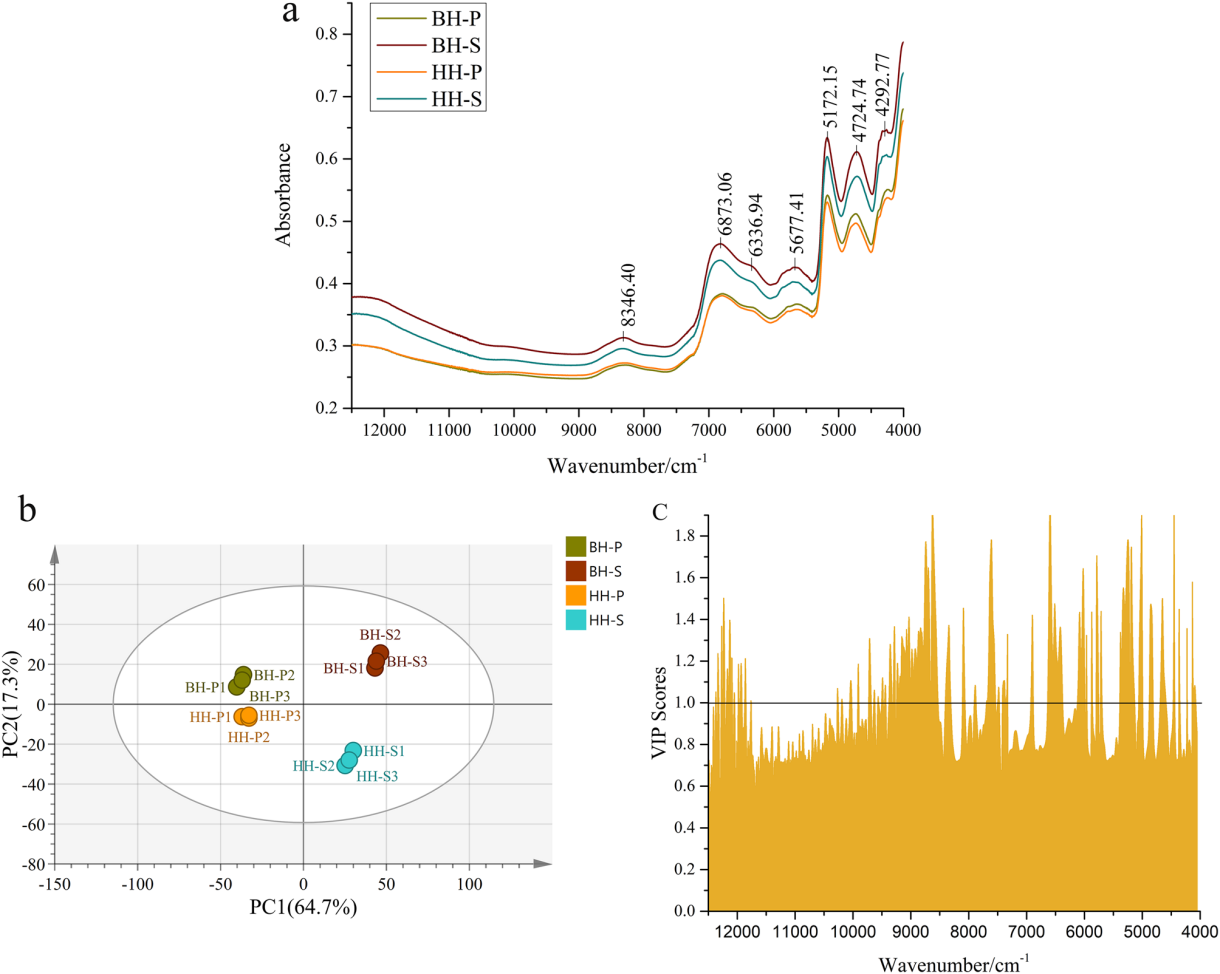
The average spectra (**a**), PCA score plot (**b**), and feature variables based on VIP method (**c**) from NIRS data.

The overlapping raw spectra showed some similar characteristic peaks and different absorption intensities, indicating differences in the accumulation of compounds in HH and BH (Fig. 5a and S4). The band 4500–4000 cm^−1^ was attributed to C–H telescopic vibration in CH, CH2, CH3 and CH═CH; the band 5500–4200 cm^−1^ may be the combination of the second overtone of C–H and O–H; peak at 7000–5500 cm^−1^ represented the stretch of C–H and O–H; band at 9000–7500 cm^−1^ may be related to the telescopic modes of second overtone in O–H and C–H^45–48^. These bonds are abundant in terpenes, aliphatic groups, phenols and carbohydrate. However, the peak position and peak shape of different varieties were similar, which was difficult to distinguish by the original spectrum, so pattern recognition method was needed in the next analysis.

NIRS data was subjected to multivariate analyses so that it could be visualized. Figure 5b showed the PCA score plot derived from NIRS data set. PC1 (64.7%) and PC2 (17.3%) explained 82.0% of the variation. The R^2^ value was 0.903 and the Q^2^ value was 0.819. The PCA plot could reveal overall differences of tissues of HH and BH samples from different varieties. BH, HH, pericarps and seeds were clearly distinguished in two principal components, indicating obvious discriminations among different groups. Although the classification models established by GC–MS and NIRS data were suitable for the discrimination of HH and BH, PCA separation results based on spectral data and PCA model established by metabolites presented some differences in the fraction of the first two principal components, which could be explained by the difference in chemical information detected in the two analytical methods. NIRS reflected the characteristics of chemical bonds and functional groups in organic compounds, mainly hydrogen-containing group information (C–H, O–H, N–H). PLS-DA model was subsequently conducted using spectral data to screen the important spectral regions of the classification model. The results showed that the accuracy of the PLS-DA classification model (R^2^ 99.0% and Q^2^ 96.2%) based on all data reached 100% without overfitting (Fig. S5). In order to verify the reliability of the classification model, two-thirds training sets and one-third test sets of samples were divided to build PLS-DA model. This model explained 84.5% of the variation, and the R^2^ value was 0.995 and the Q^2^ value was 0.961 (Fig. S6). Eventually, both the training set and the test set of the model reached 100% accuracy without overfitting, disclosing PLS-DA had excellent prediction capacity and more applicative in distinguishing BH and HH and their different tissues (Fig. S7). As can be seen from the Fig. 5c that the important classification variables were concentrated in the bands of 9384–8500 cm^−1^, 6629–6383 cm^−1^, and 5395–5127 cm^−1^. The band around 12,000 cm^−1^, 7500 cm^−1^ and 6000 cm^−1^ also showed some significant classification variables. These variables may be caused by accumulated differences in chemical composition.

## Conclusions

In this study, GC–MS untargeted metabolomics and NIRS was firstly used to analyze and discriminate HH and BH with comparable phenotypes and metabolic differences. NIRS was regarded to be an effective way to distinguish different varieties and tissues due to it has the advantages of non-destructive, easier to operate, less expensive and efficient. The results of metabolite analysis showed that there were significant differences in the metabolite expression levels between HH and BH, especially in the main components including 1,8-cineole, α-phellandrene, (E)-2-decenal, (−)-β-pinene, (E)-2-octenal, 1-octanal, D-limonene, and decanal, so it was not recommended to substitute each other. On this basis, 7 metabolites with specific expression including β-selinene, decamethylcyclopentasiloxane, (E,Z)-2,6-dodecadienal, (E)-2-hexenal, (E)-2-decenal, isogeranial, 1,8-cineole and β-cubebene were screened as potential differentiated biomarkers. KEGG pathway analysis showed that the synthesis of terpenoids was the most important metabolic pathway between the two fruits, presumably causing to metabolic discrepancy and species-specific phenotypes. Overall, this work provides a data basis for the identification of specificity and tissue differences between HH and its related species and potential marker compounds for the species differentiation. At the same time, it preliminarily opens a new direction to HH and its related species in the search for secondary metabolites, molecular markers related to variability and response to stress and bioactive compounds.

## Supplementary Information

Supplementary Information 1.

Supplementary Information 2.
