# Supplementary material for: Comparison of metabolites and variety authentication of Amomum tsao-ko and Amomum paratsao-ko using GC–MS and NIR spectroscopy
Source: Sci Rep. 2021 Jul 26;11:15200. doi: 10.1038/s41598-021-94741-0 (PMC8313684; doi:10.1038/s41598-021-94741-0)
Supplement: Supplementary file 2 — Supplementary Information 2. [file 41598_2021_94741_MOESM2_ESM.docx]

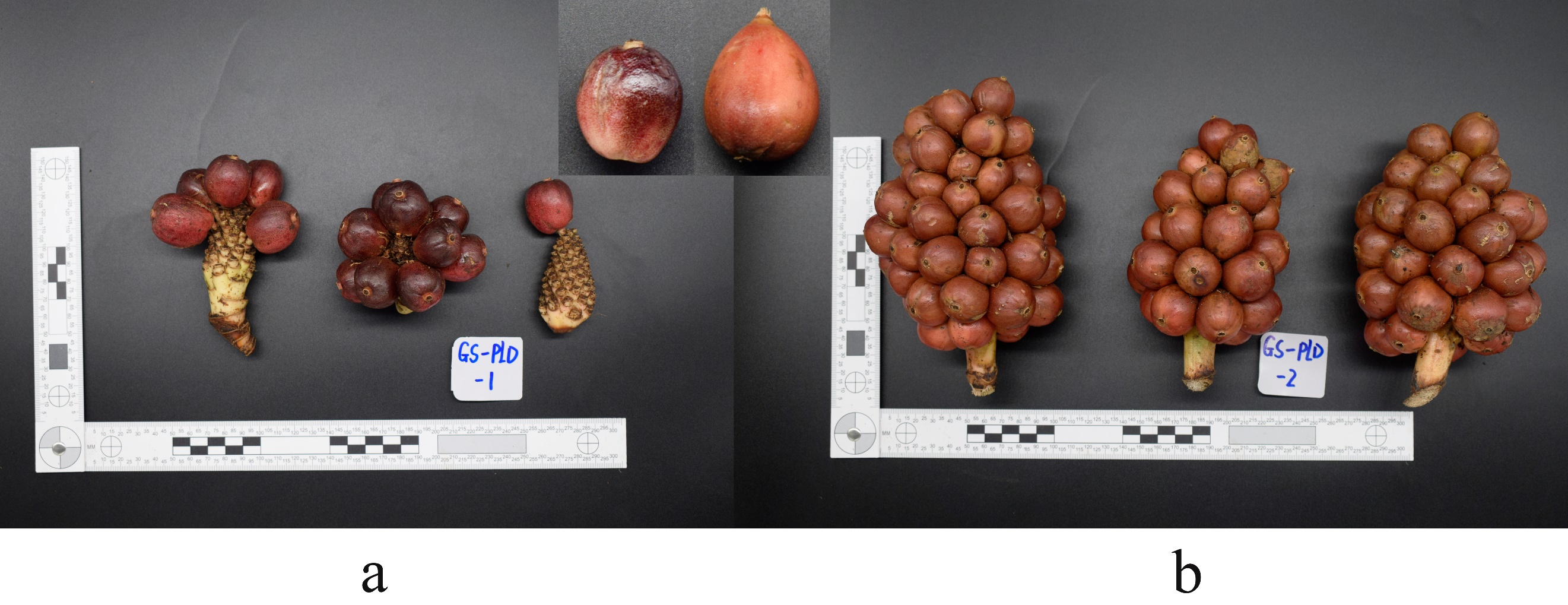


**Figure S1.** Phenotypes of two cultivars. (a) *A. paratsao-ko*; (b) *A. tsao-ko*.


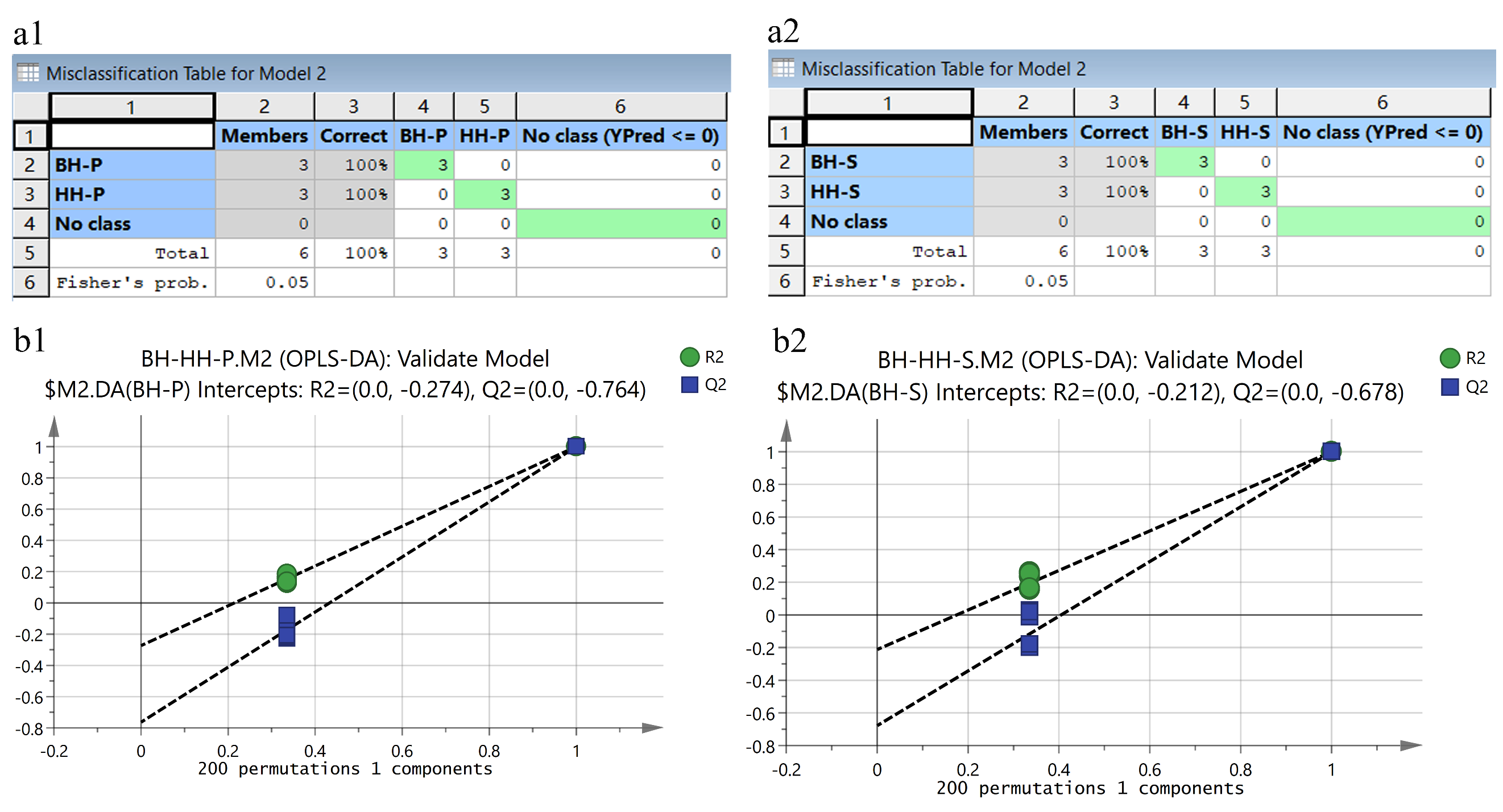


**Figure S2.** The confusion matrix (a) and permutation test (b) in OPLS-DA model of the pericarps (a1 and b1) and seeds (a2 and b2) based on GC-MS data.


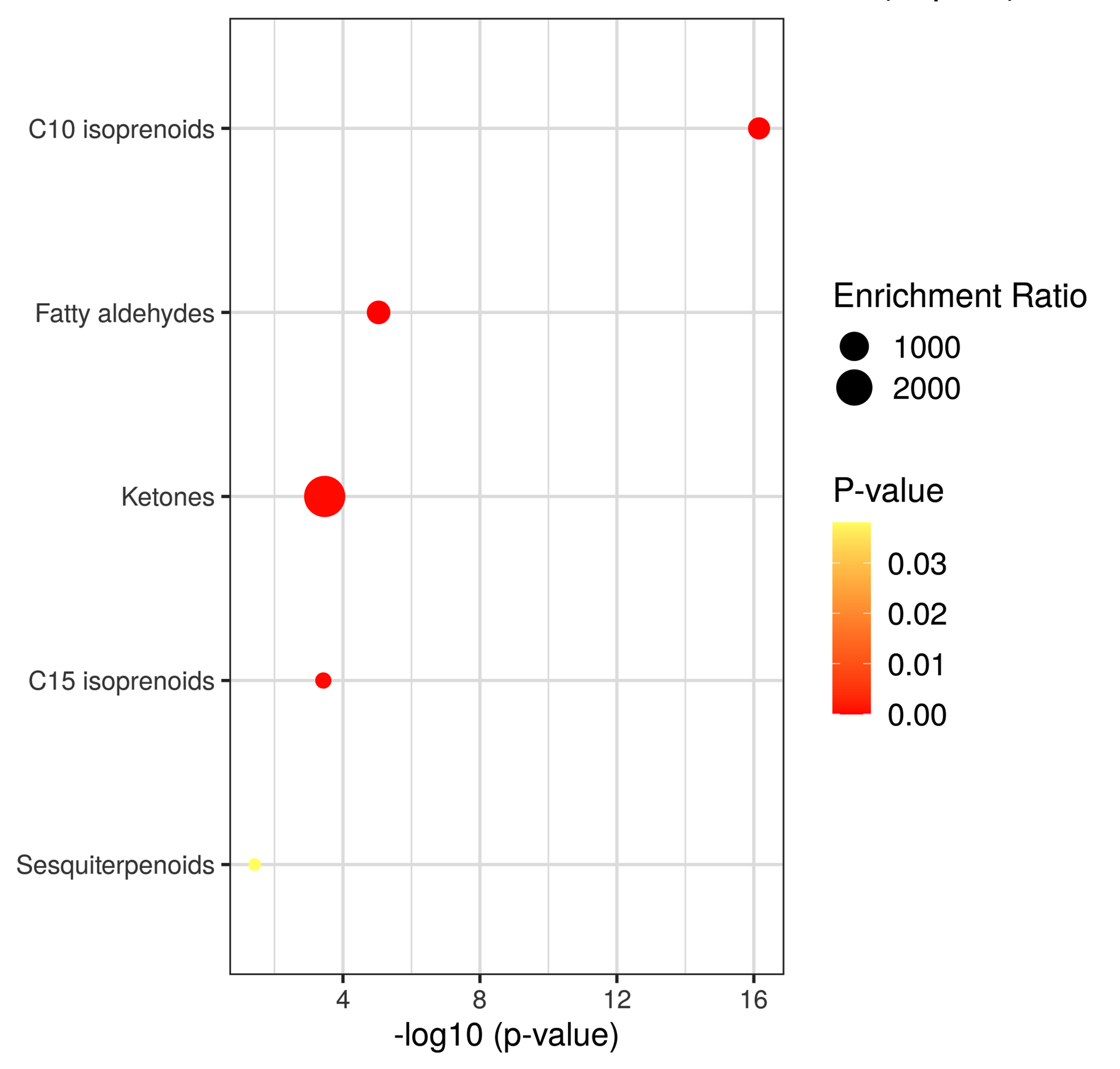


**Figure S3.** Enrichment analysis of differentially expressed metabolites and specifically expressed metabolites.


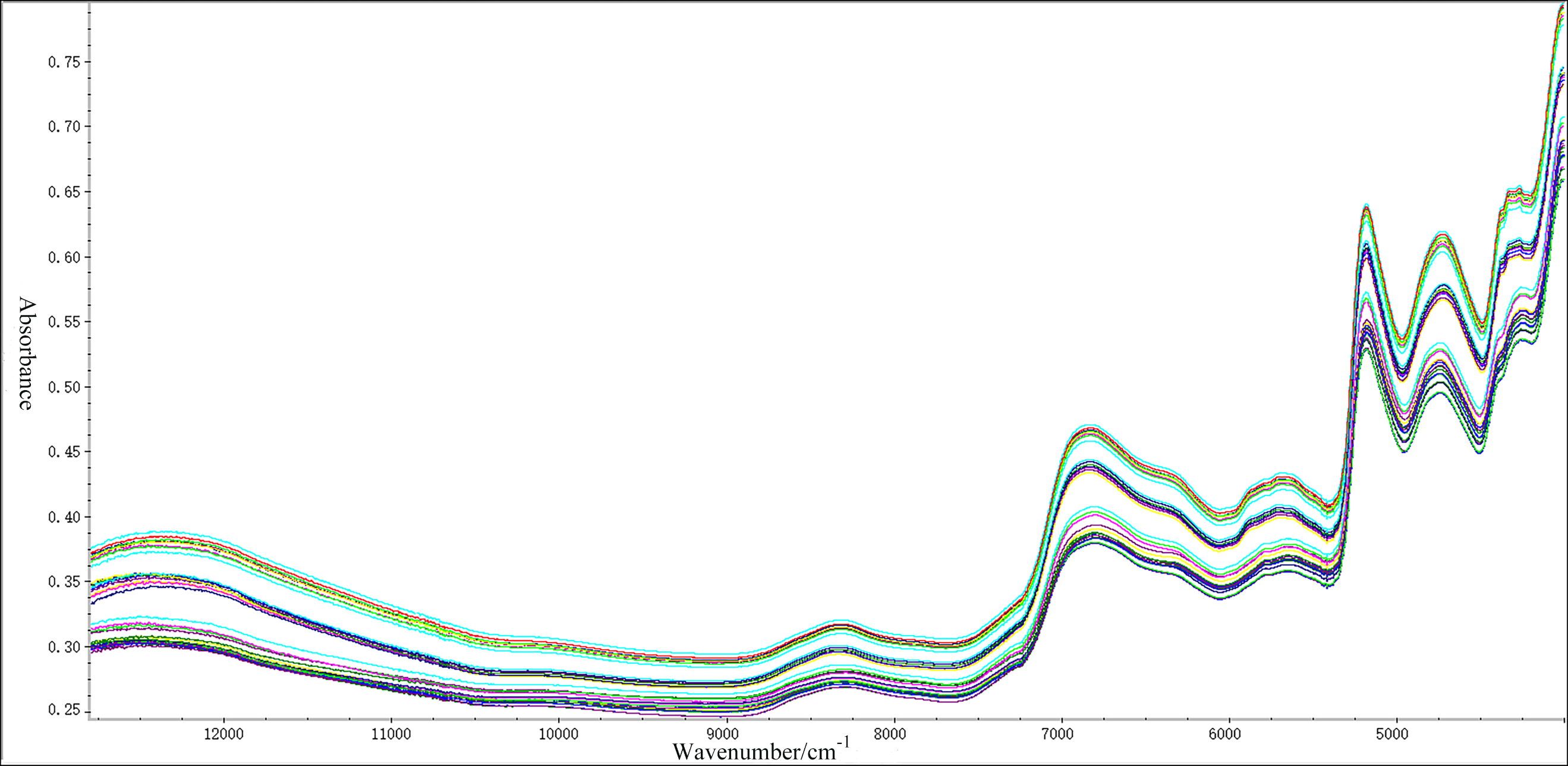


**Figure S4.** Raw NIRS of *A. tsao-ko* and *A. paratsao-ko*.


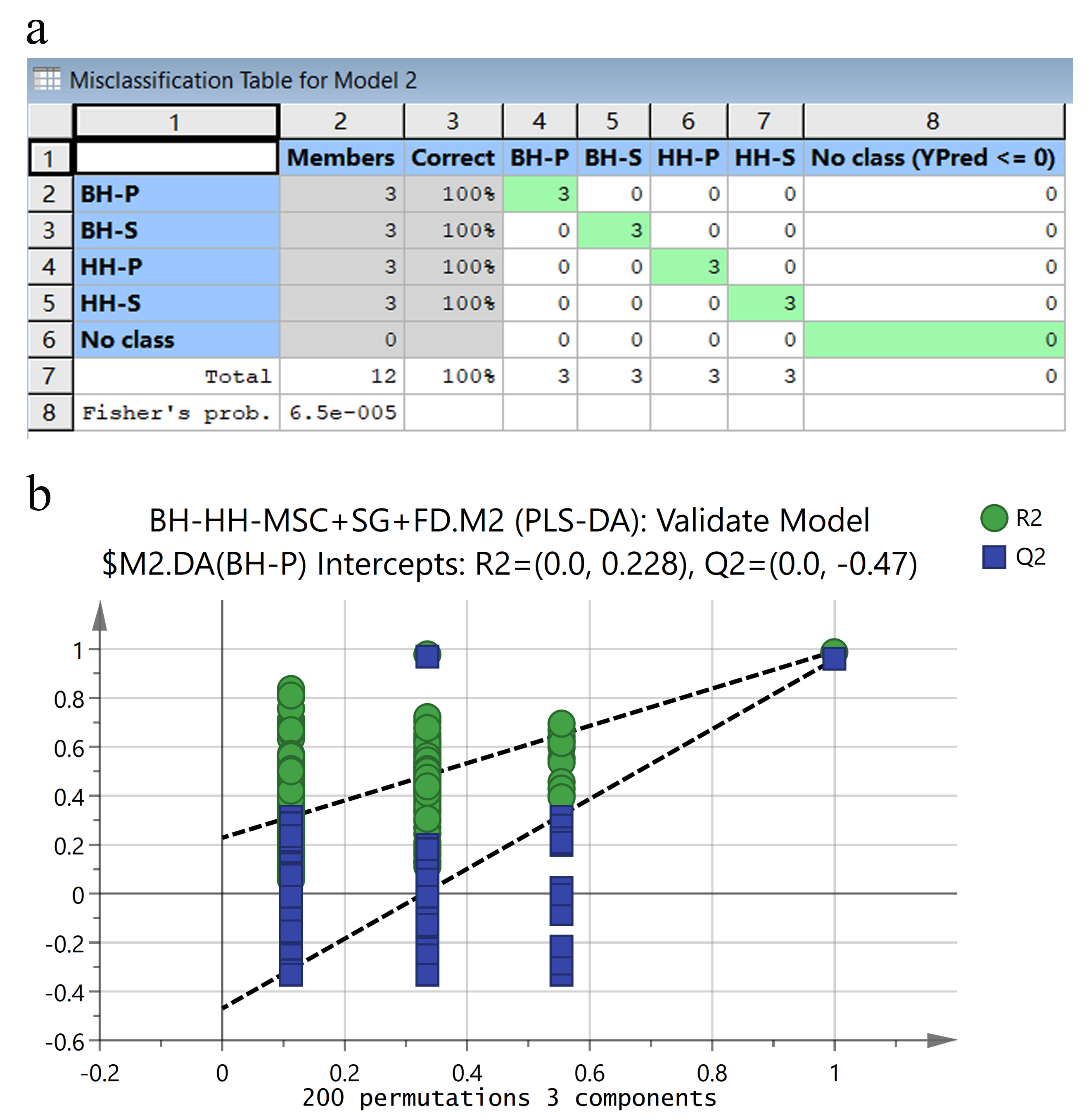


**Figure S5.** The confusion matrix (a) and permutation test (b) in PLS-DA model based on NIRS data.


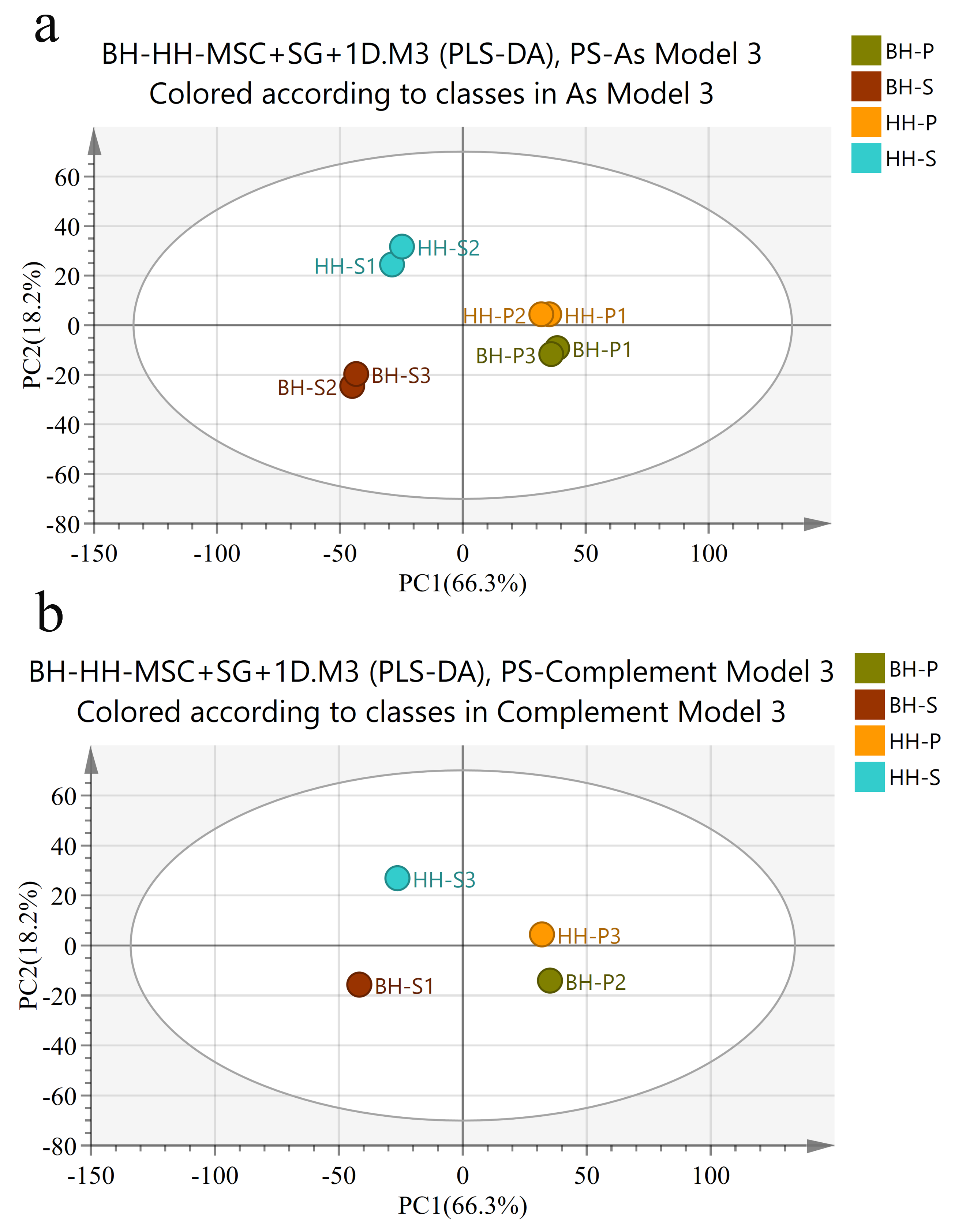


**Figure S6.** PLS-DA score plot of the training set (a) and test set (b) based on NIRS data.


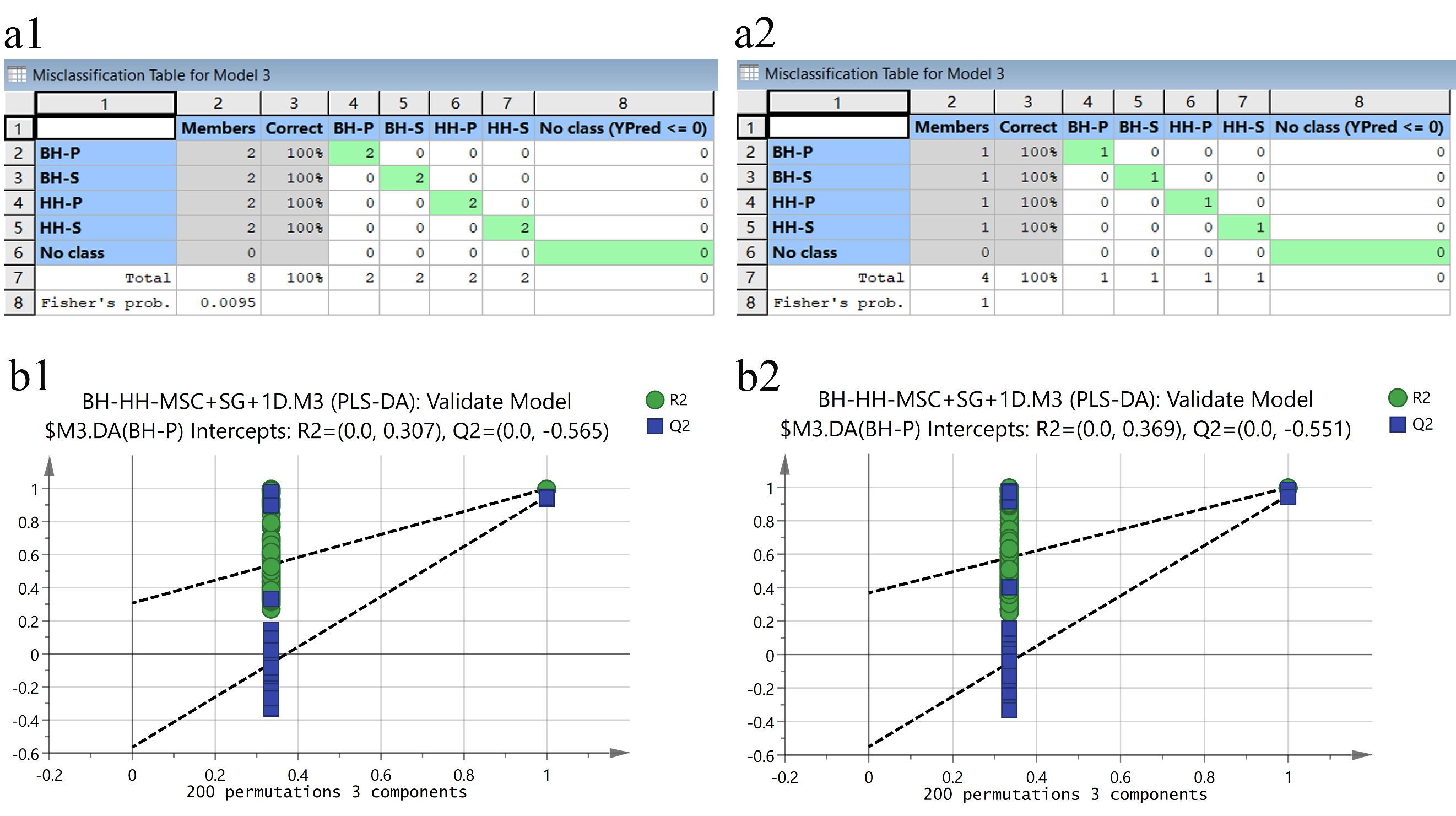


**Figure S7.** The confusion matrix (a) and permutation test (b) in PLS-DA model of the training set (a1 and b1) and test set (a2 and b2) based on NIRS data.

**Table 2S.** The relative content of major differential metabolites and potential distinguishing biomarkers between *A. tsao-ko* (HH) and *A. paratsao-ko* (BH).

| No. | Name | CAS | RT (min) | Relative content (%) | | | |
| --- | --- | --- | --- | --- | --- | --- | --- |
|  |  |  |  | BH-P | HH-P | BH-S | HH-S |
| Terpenoids | | | | | | | |
| 1 | Linalool | 78-70-6 | 21.80 | 6.74±0.160 | 1.20±0.176 | 0.236±0.0102 | 0.164±0.0276 |
| 2 | α-Phellandrene | 99-83-2 | 10.80 | 5.26±0.355 | 2.69±0.0917 | 3.90±1.03 | 15.1±2.75 |
| 3 | (Z)-Citral | 106-26-3 | 24.97 | 3.76±0.182 | 3.91±0.179 | 2.63±0.128 | 4.12±0.658 |
| 4 | Geranial | 141-27-5 | 26.13 | 4.96±0.254 | 6.00±0.239 | N | 15.5±2.39 |
| 5 | 1,8-Cineole | 470-82-6 | 12.43 | N | N | N | 19.3±13.95 |
| 6 | Tricyclene | 508-32-7 | 5.70 | N | 0.00470±0.00413 | N | 0.00805±0.00163 |
| 7 | Perillene | 539-52-6 | 18.43 | N | 0.00749±0.00649 | N | 0.00522±0.00904 |
| 8 | Sabinene | 3387-41-5 | 9.36 | 0.717±0.0259 | 2.87±0.171 | 1.16±0.0520 | 3.61±0.653 |
| 9 | D-Limonene | 5989-27-5 | 11.91 | 4.99±0.176 | 0.885±0.768 | 4.61±3.30 | 0.652±0.585 |
| 10 | β-Ocimene | 13877-91-3 | 13.15 | 6.54±0.0286 | 4.73±0.160 | 2.34±0.212 | 4.09±0.743 |
| 11 | (-)-β-Pinene | 18172-67-3 | 9.01 | N | 17.6±0.788 | 0.485±0.0355 | 1.72±0.311 |
| 12 | Isogeranial | 55722-59-3 | 22.32 | N | 0.0825±0.00629 | N | 0.158±0.0260 |
| 13 | β-Cubebene | 13744-15-5 | 25.76 | N | N | N | 0.138±0.0223 |
| 14 | β-Selinene | 17066-67-0 | 25.81 | 0.343±0.0256 | N | 0.342±0.0142 | N |
| 15 | β-Copaene | 18612-33-4 | 25.74 | 0.190±0.165 | 0.113±0.106 | 1.83±0.0680 | 0.506±0.0798 |
| 16 | γ-Muurolene | 24268-39-1 | 25.13 | 0.805±1.39 | N | 0.805±1.29 | N |
| 17 | Nerolidol | 40716-66-3 | 32.54 | 3.38±0.0385 | 0.892±0.0745 | 0.834±0.0326 | N |
| 18 | Cubebol | 23445-02-5 | 30.65 | 0.0753±0.00102 | 13.6±0.844 | 2.20±1.54 | 1.51±1.06 |
| Aliphatic group | | | | | | | |
| 19 | Sulcatone | 110-93-0 | 16.13 | N | 0.0424±0.00353 | N | 0.0139±0.0240 |
| 20 | Decanal | 112-31-2 | 20.53 | 8.73±0.160 | 0.351±0.0116 | 8.53±0.144 | 0.0349±0.00533 |
| 21 | Isoprenyl alcohol | 115-18-4 | 6.98 | N | N | N | 0.00210±0.00364 |
| 22 | 1-Octanal | 124-13-0 | 14.73 | 5.56±0.250 | 0.236±0.0144 | 12.5±0.241 | 0.635±0.0987 |
| 23 | (E)-2-Decenyl acetate | 2497-23-6 | 26.25 | 1.19±0.0489 | 0.0152±0.0263 | 1.69±0.0746 | N |
| 24 | (E)-2-Octenal | 2548-87-0 | 18.67 | 6.68±0.112 | 1.30±0.0538 | 16.8±0.417 | 3.41±0.531 |
| 25 | (E)-2-Decenal | 3913-81-3 | 24.26 | N | 19.5±0.295 | N | 8.72±1.33 |
| 26 | (E)-2-Hexenal | 6728-26-3 | 12.48 | 0.310±0.0540 | N | 0.533±0.0238 | N |
| 27 | 2,4-Dodecadienal | 21662-16-8 | 32.29 | 0.0350±0.000464 | N | 0.0220±0.00215 | N |
| 28 | (E,Z)-2,6-Dodecadienal | 21662-13-5 | 29.50 | 0.334±0.0251 | N | 0.0467±0.0805 | N |
| others | | | | | | | |
| 29 | Acetaldehyde | 75-07-0 | 1.98 | 2.12±2.07 | 0.324±0.171 | N | 0.164±0.0990 |
| 30 | Decamethylcyclopentasiloxane | 541-02-6 | 11.38 | 0.0970±0.0106 | N | 0.0230±0.00267 | N |

Average ± standard deviation. BH-P represents the pericarp of *A. paratsao-ko*, BH-S represents the seed of *A. paratsao-ko*, HH-P represents the pericarp of *A. tsao-ko*, HH-S represents the seed of *A. tsao-ko*.
